# The Effect of Intravenous Magnesium Sulfate on Post-Operative Analgesia During Laminectomy

**DOI:** 10.7759/cureus.626

**Published:** 2016-06-01

**Authors:** Sina Ghaffaripour, Hilda Mahmoudi, Hossein Eghbal, Ashkan Rahimi

**Affiliations:** 1 Shiraz Anesthesiology and Critical Care research center, Department of anesthesiology, Shiraz University of Medical Sciences; 2 GME, Aventura Hospital and Medical Center

**Keywords:** magnesium sulfate, laminectomy, patient controlled analgesia, pca, analgesia, pain management

## Abstract

Background and Objectives:

Post-operative pain control is an important concern for both patients and physicians. Magnesium is being used as an adjuvant for anesthesia and analgesia during and after various surgeries. We aimed to investigate the effects of intravenous magnesium sulfate on post-operative analgesia after laminectomy.

Methods Materials:

In this randomized double-blind controlled clinical trial, we enrolled 40 adult patients aged 18-60 with American Society of Anesthesiologists (ASA)  Class I-II who were candidates for elective laminectomy. The patients were randomly assigned in two control groups and were similarly anesthetized. In the case group, after the induction of anesthesia, a loading dose of magnesium sulfate (30 mg/kg) was administered within five to 10 minutes followed by a maintenance dose of 10 mg/kg/hr up to the end of the surgery; while, the patients in the control group received the same volume of saline. After the surgery, all patients received a patient-controlled intravenous analgesia (PCA) pump containing morphine. The first time of using PCA, the amount of consumed morphine during the first 24 hours, and pain score were recorded at 6,12,18 and 24 hours in the post-operative period.

Results:

There was no significant difference between the two groups with respect to the amount of morphine consumed in 24 hours after the surgery (P value =0.23), the first time of using of PCA pump (P value =0.79) and pain intensity (P value=0.52).

Conclusion:

The infusion of Magnesium Sulfate during laminectomy had no effect on patients’ pain and opioid requirement during the first 24 hours after the surgery.

## Introduction

Surgical pain is caused by inflammation of damaged tissues or direct injury to nerve cells. Post-operative pain control is an important concern for both patients and physicians. It helps in early ambulation after surgery, increases patient satisfaction and reduces the costs of hospitalization by shortening the length of hospital stay. Factors which are associated with post-operative pain can be generally classified into two groups; patient-related and surgery-related factors. Patient-related factors include previous pain experiences, social, cultural and psychological status, as well as genetic and sexual factors. However, surgical factors include the type of anesthesia and surgical technique including ability to diagnose and avoid nerve damage if possible [1].

While, the stimulation of pain fibers, which send nerve signals to brain, is the same in all humans, pain perception and control may vary from one population to another [2]. The reason is perception, expression and control of the pain are learned behavior and culturally specific. Pain perception depends on a combination of emotional, cognitive and sensory components. Patient’s expression is the most important indicator of pain intensity and perception. Despite the fact that the mechanism of pain sensation is complex and influenced by multiple factors, expression of distress may differ across cultural groups [3].

There are many techniques for pain management after surgery including systemic analgesia such as opioid and non-opioid drugs and regional techniques like neuraxial and peripheral blocks. Physician chooses an appropriate analgesic regimen for each patient with respect to the advantages and disadvantages of each technique and the patient’s preference. Magnesium (Mg) is an essential cation that its homeostasis plays an important role in the normal function of the body. Magnesium is a calcium channel blocker and non-competitive antagonist of *N*-methyl-D-aspartate receptor with analgesic properties. Mg is used as an adjuvant for analgesia during and after various surgeries [4]. Not only it is cheap and available but it also causes less respiratory depression compared with opioids.

While analgesic effects of Mg have been confirmed by previous studies [5-8], some other studies failed to prove such effects [9]. Furthermore, some studies have pointed out the role of cultural and racial differences in the pain expression and perception [10].

Despite the existence of numerous factors that cause radiculopathy, inter-vertebral disc herniation is considered as the common cause of lumbo-sacral radiculopathy. Although, the factors causing disk herniation are poorly known, major causes are early degenerative changes that lead to drying nucleus populous and cracking the margins of annulus fibrosus. However, careful patient selection is the key of success in surgical interventions [11].

We aimed to investigate the effect of intravenous Magnesium Sulfate on the postoperative analgesia in the patients undergoing laminectomy. 

## Materials and methods

This study was approved by the Institutional Ethics Committee of Shiraz University of Medical Sciences and written informed consent was obtained from all patients. The study was registered in Iranian Registry of Clinical Trials (IRCT no: IRCT2015012019470N12). In this randomized double-blinded controlled clinical trial, we enrolled 40 ASA I-II adult patients, aged between 18 and 60 years undergoing elective laminectomy in Nemazee and Shahid Chamran Hospitals, Shiraz, southwest Iran.

Exclusion criteria were cardiovascular, renal or hepatic dysfunction, cardiac conduction abnormalities, history of magnesium or calcium channel blockers consumption, drug abuse and allergy to magnesium sulfate or other study drugs. The patients with coagulation disorders and emergency surgery were also excluded from the study.

The patients were randomly assigned in two groups, twenty patients in each group, to receive Mg sulfate or Saline. We used block randomization method and sealed envelope to assign patients to groups.

All the patients were monitored by noninvasive blood pressure, heart rate, pulse oximetry, electrocardiogram and end tidal CO2. The two groups were similarly anesthetized with midazolam 0.03 mg/kg, fentanyl 3µg/kg, morphine 0.1 mg/kg, thiopental 5 mg/kg and 0.6 mg /kg atracurium. In the case group, after the induction of anesthesia, a loading dose of magnesium sulfate (30 mg/kg) was administered within 5 to 10 minutes followed by a maintenance dose of 10 mg/kg/hr up to the end of the surgery; while, the patients in the control group received the same volume of saline. Anesthesia in all patients was maintained using nitrous oxide, oxygen, and 1-1.5 minimum alveolar concentration (MAC) of isoflurane. The duration of operation was similar in both groups and less than ninety minutes regarding the operation, which was simple elective laminectomy.

In case of a patient with hypotension (mean arterial pressure ˂55 mmHg), 5 mg ephedrine was to be injected so that the mean arterial pressure could reach higher than 55 mm Hg. However, none of our patients experienced hypotension less than the specified limit. If arrhythmia occured in any of the patients during operation, proper treatment had to be performed and the patient would be excluded from the study. The magnesium sulphate and anaesthetic agent infusions were discontinued at skin closure. The relaxant effect of magnesium sulfate was also considered. Then, train of four (TOF) monitoring was used to monitor the muscle relaxation during the surgery and patients were extubated at the end of the operation based on the TOF > 0.9 to ensure the complete reversal of relaxants by neostigmin.

After the surgery all patients received a patient-controlled intravenous analgesia (PCA) pump (Autofuser; South Korea; reservoir: 275 ml; bolus dose: 2 ml; lock-out time: 15 minutes) containing morphine based on their need for analgesia. Accordingly, if the patients required analgesia, he/she could inject a bolus dose of 1 mg morphine with a 15-minute lockout interval. However, in recovery room, if VAS was more than 4 , one mg morphine was injected every five minutes until VAS reaches less than 4 and if VAS was more than 8, two mg morphine was injected every five minutes by the pain nurse until VAS reaches less than 7, then the protocol continued to reach VAS<4.

The first time of PCA analgesic consumption and the total amount of consumed morphine at 24 hours after operation were recorded. Pain scores at 6, 12, 18 and 24 hours after operation was measured using a 0–100 mm visual analogue scale (VAS) starting from zero (no pain) to 100 (worst pain imaginable). An acute pain service nurse who was blinded to group assignments collected all the information.

The primary outcome of the study was total amount of postoperative PCA morphine consumption. We considered first time of PCA analgesic consumption and pain score as secondary endpoints.

Sample size of 20 patients in each group was based on an estimated post-operative 24h PCA morphine consumption of 20.10 (SD=3) mg in control group [8] and a minimum clinically significant difference between the groups of 3 mg, using two sided alpha of 0.05 and power of 90%.

The Mann-Whitney U test was used to compare total amount of postoperative PCA morphine consumption, first time of PCA analgesic consumption in two groups. Repeated measures ANOVA was used to compare postoperative VAS. If there was a statistical difference (P=0.05) between the two groups by repeated measures ANOVA, the Mann-Whitney U test was used to compare the data at each time point. Values are expressed as counts or means (SD). Two sided P-values of 0.05 were considered statistically significant.

## Results

Forty patients were equally assigned in two groups. One patient from the control group dropped out of the study due to post operation dyspnea and chest pain. There was no difference between the two groups with regards to female/male ratio (8:11 vs. 8:12 in magnesium and placebo group respectively) and age (44.7 yr vs. 43.5 yr in magnesium and placebo group respectively).  

The mean of total amount of postoperative PCA morphine consumption was 0.7(0.08) mg/kg in placebo group and 0.59(0.04) mg/kg in Magnesium group which was not statistically different (P value =0.23).

The mean time of first PCA analgesic consumption was 3.73 (1.42) and 3.61 (1.52) hours post operatively  in the placebo and magnesium group respectively that was not significantly different (P value =0.79).

The VAS was checked every 6 hours to compare the intensity of pain. The mean of pain intensity in the control and study group was 3.89(1.19) versus 3.85(0.87) at 6 hours; 3.26(0.99) versus 3.40(0.75) at 12 hours; 2.11(0.87) versus 2.45(0.99) at 18 hours and 1.37(0.68) and 1.20(0.83) at 24 hours after the surgery. No significant differences were found in VAS score trend by time between the two group (p=0.52).

## Discussion

In this study we didn’t find that infusion of magnesium sulfate can provide a clinically important reduction in opoid consumption and pain severity in patients undergoing laminectomy in the first 24 hours postoperatively. In consistency with our results, Bahatia and colleagues studied the effects of Mg infusion on analgesia during cholecystectomy and reported no significant decrease in the amount of consumed morphine [12]. In contrast to our findings, previous studies reported the analgesic effect of magnesium sulfate. Kogler examined the analgesic effect of Magnesium sulfate on patients who underwent thoracotomy procedure. He observed that intraoperative fentanyl consumption was decreased significantly in Magnesium treated group compared to control group. However, there was no difference in pain intensity at 48 hours after surgery [13]. Bilir and colleagues also examined the analgesic effect of epidural Magnesium sulfate on patients undergoing elective hip replacement and revealed that less fentanyl was consumed for analgesia in the patients receiving magnesium sulfate [14].

In a meta-analysis twenty five trials comparing magnesium with placebo were evaluated. They concluded that peri-operative intravenous magnesium reduces opioid consumption, and to a lesser extent, pain scores, in the first 24 hour postoperatively [15].

One potential explanation of this controversy between our findings with previous studies is difference in patient population. In the meta-analysis above almost 50% of patients underwent abdominal surgery which is different from laminectomy. In our study, patients who are candidate for laminectomy due to disc herniation are mostly complaining of back and radicular pain that has suffered them for months or even years. Therefore, careful patient selection is the success key in the treatment of the patients undergoing laminectomy. The patients who are selected carefully and well treated by the surgery could have a relief from chronic pains that they had for long time. Relieving from such pain may be effective to the extent that helps them forget the surgery pain or tolerate it easier. It can possibly be the cause of observing no differences between the Mg sulfate group and control group with respect to the morphine consumption, the first time of using PCA and pain intensity. It is observed in some RCTs [16, 17] that the VAS score has dropped by half (from 7.8 to 3.8) after discectomy in placebo group for pain control, confirming that surgery, by itself, diminishes majority of the preoperative pain perception without any other intervention. Perhaps we could derive a better conclusion if the patients were asked to rate their pain based on the VAS scale before the surgery and we could compare them with post-operative measures.

Naturally, the patients who have chronic pain and have been taking analgesics and opioids for a long time may experience opioid tolerance after operation, causing them to require increased opioid doses to achieve the desired effect [18]. But such patients were excluded from the study, we found no increase in need for morphine consumption in our patients.

Pain perception can be influenced by variety of factors such as gender, psychological, personality, genetic and ethnicity [19, 20]. Some ethnicities can tolerate pain better than others [19, 21]. In some cultures tolerating pain is considered as a pleasant or acceptable experience [22]. In both groups of our study, pain score was moderate particularly in early hours (mean VAS of 4); and the amount of injected morphine and number of PCA pump usage was not so high comparing to previous findings that partially is explained by socio-cultural  difference in our population comparing to other countries.

We recognize that there are limitations to our study. We only recruited patients undergoing laminectomy which limits the generalizability of our finding. Furthermore, the study population was not large; however the sample size was based on predefined effect size and power.

## Conclusions

In conclusion, we found that infusion of magnesium sulfate during laminectomy had no effect on the patients' pain and their need for opioid during the first 24 hours after surgery. Caution should be expressed in extrapolating this result in view of the type of surgery performed on our patients and the socio-cultural difference in our population compared to the population from western countries as this difference impacts the perception of pain. 
